# Effect of Yoga involvement on mental health in times of crisis: A cross-sectional study

**DOI:** 10.3389/fpsyg.2023.1096848

**Published:** 2023-03-22

**Authors:** Christiane Bös, Lisza Gaiswinkler, Jürgen Fuchshuber, Andreas Schwerdtfeger, Human-Friedrich Unterrainer

**Affiliations:** ^1^Institute of Psychology, University of Graz, Graz, Austria; ^2^Center for Integrative Addiction Research (CIAR), Grüner Kreis Society, Vienna, Austria; ^3^Department of Philosophy, University of Vienna, Vienna, Austria; ^4^Department of Psychiatry and Psychotherapeutic Medicine, Medical University of Graz, Graz, Austria; ^5^Department of Religious Studies, University of Vienna, Vienna, Austria; ^6^Faculty of Psychotherapy Science, Sigmund Freud University, Vienna, Austria

**Keywords:** Yoga, Yoga involvement, subjective well-being, psychological distress, sense of coherence, spirituality, COVID-19, crisis

## Abstract

**Background:**

Yoga involvement can be understood as a degree of immersion in the philosophical/spiritual teachings of Yoga. Previous research has shown a positive association between Yoga involvement and mental health. This study further investigates the effect of Yoga involvement on several parameters of psychological well-being and distress amidst a global crisis precipitated by the COVID-19 pandemic.

**Methods:**

A total sample of 246 participants (216 females; 118 Yoga versus 128 age-matched Pilates practitioners) were included in the study. *Via* an online-survey the following questionnaires were completed: the WHO-Five Well-Being Index, the Multidimensional Inventory for Religious/Spiritual Well-Being short version, the Leipzig Short Scale of Sense of Coherence, the Brief Symptom Inventory and the Yoga Immersion Scale which was adapted for the control group to Pilates Immersion Scale.

**Results:**

When controlling for occupation and psychiatric disorder, involvement in general was higher for Yoga practitioners than for Pilates controls. Furthermore, Yoga practice was associated with increased religious/spiritual well-being and decreased sense of coherence, but neither with psychological well-being nor with psychological distress. Involvement, in general, positively predicted psychological well-being, religious/spiritual well-being and sense of coherence, but there was no connection with psychological distress. Lastly, involvement mediated the positive relationship between Yoga practice and religious/spiritual well-being and suppressed the negative effect of Yoga practice on sense of coherence.

**Conclusion:**

Our findings suggest that involvement in a certain relaxation practice has a positive impact and might be a key component in understanding Yoga’s influence on mental health. Further randomized controlled research, including clinical groups, is necessary to explain how involvement changes and how it effects well-being more specifically.

## Introduction

“The world is upside down, and so am I” is how one participant of this study described their current experience during a headstand.^1^ The global outbreak of the respiratory coronavirus disease COVID-19 in March 2020, has led to an international public health emergency with profound political, sociocultural and economic implications. The measures taken to contain the pandemic—lockdowns and restrictions in occupational, social, recreational and cultural life—are related to increased chronic stress, sleep disturbances, loneliness, depression, and anxiety (Röhr et al., 2020; Traunmüller et al., 2020). Life satisfaction and the experience of positive feelings are diminished (Zacher and Rudolph, 2021). The pandemic is thus not only a global health crisis but a mental health crisis. The length and scope of the ongoing situation can be understood as a collective trauma (Holman et al., 2022) that is further exacerbated by the economic recession, the recent Russian invasion of Ukraine in February 2022 and consequences of climate change that are “approaching irreversibility” (IPCC, 2022, p. 9). A pressing issue is, therefore, to help people find strategies to cope with the enduring stress throughout challenging times. A discipline that combines exercise with relaxation and could, hence, serve as a suitable coping strategy is the practice of Yoga.

According to Yoga tradition, practitioners strive for greater balance, reduction of suffering and spiritual development (Michelis, 2005). This pursuit is facilitated through a holistic system of techniques, including postures, breathing methods, meditation, self-reflection, and ethical guidelines (Taimni, 2005). Yoga techniques support and build upon each other to achieve a deeper level of consciousness (Taimni, 2005).

One of the underlying mechanisms that encompasses this deeper level of consciousness, with which Yoga practice might mitigate the psychological impact of the crisis, could be the level at which people immerse themselves in their practice—their *involvement*. Yoga involvement is a measure of how central the identity of as Yoga practitioner is to an individual’s self-concept (Gaiswinkler and Unterrainer, 2016). It is derived from Huber’s (2004) centrality scale for measuring religiosity that is based on Kelly’s (1955) classic Personal Construct Theory. Accordingly, a high level of involvement means that the identity as a Yoga practitioner plays a central role in their personal construct system, which corresponds to an increased intrinsic motivation (Gaiswinkler et al., 2015). This depth of involvement into the practice might help to explain why and how yoga practice fosters different aspects of mental health.

An ever-growing body of research attests to Yoga’s psychological benefits, including an increase of overall well-being (Woodyard, 2011; Trulson and Vernon, 2019), possibly because different aspects of the practice (e.g., physical exertion, breathwork, meditation) strengthen various psychological resources such as mindfulness, body consciousness, self-transcendence, social connectedness and spirituality which in turn foster positive emotional experiences (Park et al., 2020). Despite these associations, research is not as extensive as one might expect (Hendriks et al., 2017) and especially research concerning the exact mechanisms of Yoga is limited. Additionally, there have been methodological concerns, including a shortage of randomized controlled trials, small sample sizes, and difficulty in fully capturing the complexity of the practice (Jeter et al., 2015). A systematic review found that Yoga practice increased positive affect in some studies but had no significant influence on positive affect in others (Domingues, 2018). Throughout COVID-19 lockdown, Yoga practitioners experienced higher well-being than both non-practitioners and practitioners of other spiritual practices (Sahni et al., 2021) and an online Yoga intervention was able to increase participant’s well-being when compared to a waitlist control group (Wadhen and Cartwright, 2021). Aside from the general effect of Yoga, Yoga involvement is associated with feelings of happiness (Gaiswinkler et al., 2015) and with harmonious passion (Domingues and Carmo, 2021).

Religious/spiritual well-being (RSWB) has been defined as “the ability to experience and integrate meaning and purpose in existence through a connectedness with self, others, or a power greater than oneself” (Unterrainer et al., 2011, p. 360). A systematic review discovered that Yoga seems to be associated with spirituality and more specifically with spiritual aspirations, a search for wisdom, an integrative worldview, a sense of meaning and peace, faith, hope and compassion (Csala et al., 2021). Moreover, It has been found that higher involvement in the Yoga practice is related to an increased overall RSWB and to all of its subscales (Gaiswinkler and Unterrainer, 2016). Correspondingly, an intense practice might positively influence a Yoga practitioner’s spirituality, mood and mindfulness (Büssing et al., 2012a) and a regular practice might be necessary to reap the spiritual benefits of Yoga (Csala et al., 2021).

Increasing and maintaining well-being is also the subject of Antonovsky’s Salutogenesis model (1987, 1991) that encompasses preconditions and promotion of health Sense of coherence (SOC) is the core concept of Salutogenesis and refers to the feeling that there is predictability and meaning in life, that things will turn out as they should (Antonovsky, 1988). It comprises three components: Understandability, Manageability and Meaningfulness. A pronounced SOC is strongly related to various predictors of health (specifically mental health) and of life satisfaction (Eriksson and Lindström, 2006, 2007). Originally, Antonovsky proposed that SOC does not change after it has been developed in youth, however, current research suggests that SOC can shift over time (Feldt et al., 2003) and probably even be modified by religious/spiritual interventions (Jeserich, 2013). Since the beginning of the COVID-19 pandemic, people reported feeling a loss of control, freedom and predictability (Zhai and Du, 2020; Statz et al., 2022). Since SOC captures these states, it would be useful to know whether Yoga involvement has an impact on them. Although literature on the relationship between SOC and Yoga is limited, promising findings are emerging. A small study (*N* = 15) was able to show that Hatha-Yoga practitioners have a higher SOC than non-practitioners (Gwiaździński et al., 2017). Furthermore, a 2-week Mindfulness-based meditation therapy (which included breathing exercises, Yoga, exercise, and meditation) significantly increased SOC and, particularly, sense of purpose in a group of nurses (Ando et al., 2011). The association between Yoga involvement and SOC has yet to be investigated.

The relationship between Yoga practice and psychological distress is complex. Yoga practice has been found to alleviate psychological symptoms (Büssing et al., 2012b). However, Yoga practitioners also displayed higher rates of depression and anxiety than a representative sample (Birdee et al., 2008) and had more frequent contact with mental health services (Fouladbakhsh and Stommel, 2010). An possible explanation could be that particularly vulnerable people might be more likely to pursue Yoga to soothe their symptoms (Orlygsdottir et al., 2021) because treatment of mental conditions is often given as a reason to why people practice Yoga (Birdee et al., 2008). COVID-19 containment measures are associated with chronic stress, sleep disturbance, loneliness, depression and anxiety (Röhr et al., 2020). Causes of these symptoms include social isolation, financial hardships and loss of control (Pietrabissa and Simpson, 2020). People in quarantine are often plagued by insomnia (Röhr et al., 2020). Yoga may provide relief, as a correlation has been found between regular Yoga practice and improved quality of sleep (Wang et al., 2020). This may be due to the exertion during Yoga postures along with improved tissue blood flow and oxygenation through breathing exercises (Bankar et al., 2013). Besides sleep problems, stress levels increased under COVID-19 related restrictions. Yoga reduces stress by regulating the sympathetic nervous system (Pascoe et al., 2017). In correspondence to this, a meta-analysis found a positive effect of Yoga on both depressive symptoms and depressive disorders (Cramer et al., 2013). Other meta-analyses showed evidence of Yoga easing anxiety (Li and Goldsmith, 2012; Cramer et al., 2018). In addition, not only Yoga but also a high Yoga involvement has been found to predict lower levels of psychological distress, especially for anxiety and depression symptoms (Gaiswinkler and Unterrainer, 2016), and may help to buffer the negative effects of the current crisis and alleviate psychological symptoms.

### Research aims and hypotheses

The present study seeks to assess the role of involvement in the effect of Yoga on various aspects on mental health: psychological well-being, RSWB, SOC and psychological distress. For this purpose, we conducted a cross-sectional online survey that compares Yoga practitioners to Pilates controls. Due to the spiritual and meditative nature of Yoga, we expect (1) that the involvement in the Yoga group is higher than in the Pilates group. In line with previous research, we assume a practice with a spiritual and meditative dimension should be more deeply anchored in the personality construct of the practitioners than a practice that is mainly physical (Gaiswinkler and Unterrainer, 2016).

Additionally, we hypothesize that (2) a higher involvement in the respective practice is associated with an increased general and spiritual well-being (Gaiswinkler and Unterrainer, 2016), an increased SOC, and decreased psychiatric distress. Given the expected positive association between group membership (Yoga versus Pilates group) with involvement and the expected positive association between involvement and psychological well-being, RSWB and SOC and the expected negative association between involvement and psychological distress, we hypothesize (3) a mediation effect in which the positive association of Yoga with psychological well-being, RSWB and SOC and the negative association of Yoga with psychological distress occurs *via* increased involvement. In a nutshell, we propose that involvement might be a key mechanism through which Yoga practice promotes mental health.

The current study also has an exploratory component that investigates potential correlates for increased involvement. Since increased Yoga involvement corresponds to a high centrality of Yoga in the self-concept (Gaiswinkler and Unterrainer, 2016), motivations for Yoga practice that are more central to the practitioner’s identity should also lead to an increased involvement in the Yoga practice. For example, people who practice Yoga for spiritual reasons or for self-discovery should be more immersed in their practice than people who want to lose weight.

## Methods

### Participants and procedure

In total, 424 participants were recruited online *via* convenience sampling (required sample size estimation based on Fritz and MacKinnon, 2007). For this purpose, a link to the study was posted to practice-specific message boards, e-mailed to Yoga and Pilates studios, and distributed *via* the e-mail list of the University of Graz over 4 weeks. To increase the validity of the results, only subjects who did their practice of choice a total of at least five times and the practice of the opposing condition no more than five times since the beginning of the pandemic could partake (“Have you practiced Pilates/Yoga five or more times since the beginning of the COVID-19 Pandemic?”). In addition, only participants located in either Germany, Austria, or Switzerland were included, since these countries’ epidemiological demands throughout the COVID-19 pandemic and their outcomes were comparable (Desson et al., 2020).

Participants were assigned to one of two conditions determined by their preferred type of practice (Yoga or Pilates). The study itself comprised an online questionnaire in which informed consent was obtained first, followed by screen-out questions, socio-demographic questions, crisis-, health-, and practice-related questions and questionnaires. The survey took about 10 min to complete. As compensation, participants could enter a raffle for one gift certificate (€50). In addition, psychology students at the University of Graz could receive class credit for their participation. Approval for the study was granted by the Ethics Committee of the University of Graz.

Thus far, Yoga involvement has only been examined with a heterogeneous control group of various gymnastic styles (Aerobics, pelvic floor exercises, gymnastics for the spinal column and Pilates) in which the Yoga group was split into three subgroups: low, medium, and high involvement (Gaiswinkler and Unterrainer, 2016). Although Yoga involvement has been defined in the same study as the “extent of personal involvement into the teachings of Yoga running on a continuum” (p. 124), no research so far investigated whether involvement predicts mental health outcomes when it is treated as a linear variable. Additionally, a thorough comparison with a more homogenous control has not been attempted yet. Pilates was chosen as the control group because active control groups are recommended (Park et al., 2014) and because Pilates is a physically similar, yet non-spiritual practice (Yashika and Vasanthi, 2019). Previous research by Gaiswinkler and Unterrainer (2016) compared the effect of Yoga involvement on different indicators of mental health but did not extend the concept of involvement to the control group. This study, therefore, compares levels of involvement between Yoga and Pilates participants.

### Psychometric assessment

Socio-demographic information such as age, gender, marital status, and religious affiliation was obtained. To determine whether the two groups were similar across all relevant contexts, we asked about participants’ circumstances during the pandemic, their health and their practice. Pandemic-related questions included system relevance of occupation, demands during the pandemic and the extent of urbanization of the participant’s residence. Health-related questions addressed overall health status, presence of physical or mental illness and additional exercise. Practice-related questions assessed length, frequency and the main reason for starting the practice.

The *Yoga Immersion Scale* (YI-S; Gaiswinkler et al., 2015) is a self-report measure that assesses the degree of involvement in the Yoga practice. The test includes 10 items answered on a 6-point Likert scale (1 = *strongly disagree* to 6 = *strongly agree*). The scale displays high internal consistency (*α* = 0.91; Gaiswinkler et al., 2015). For the control group, the scale was renamed to *Pilates Immersion Scale* (PI-S), the word “Yoga” was replaced by “Pilates,” and two of the items were secularized: “The theoretical concepts of Pilates [wisdoms of Yoga] also influence my other ways of looking at things in everyday life” and “Studying the historical sources of Pilates [wisdoms of Yoga] helps me a lot.” (see supplementary materials for the full list of items; original German language and English translation).

The *WHO-Five Well-Being Index* (WHO-5; Version II; Bech et al., 2003; German version by Singer and Brähler, 2007) is a unidimensional scale that uses five items to estimate well-being within the last 2 weeks including mood, vitality, and general interests. Items are scored on a 6-point Likert scale (0 = *at no time* to 5 = *all of the time*), with a higher total score indicating greater psychological well-being. Items include: “I have felt cheerful and in good spirits” and “My daily life has been filled with things that interest me.” The measurement instrument is characterized by very good internal consistency (*α* = 0.92) and high construct validity (Brähler et al., 2007).

The short version of the *Multidimensional Inventory for Religious/Spiritual Well-Being* (MI-RSWB 12; Unterrainer and Kapfhammer, 2014, see also Vuzic et al., 2022) is based on the MI-RSWB long version with 48 items (Unterrainer et al., 2010; see Unterrainer et al., 2011 for the full list of English items) and measures the total of RSWB. It contains 12 items which are measured on a 6-point Likert scale (1 = *strongly disagree* to 6 = *strongly agree*). Four subscales (General Religiosity, Hope, Forgiveness, Connectedness,) as well as a total RSWB score can be calculated. Example items are “I have experienced the feeling of being absorbed into something greater” and “I view the future with optimism.” The total score as well as the sub-scales were found to be sufficiently reliable (total score *α* = 0.76, *ω* = 0.77; sub-scales *α* > 0.6; Unterrainer and Kapfhammer, 2014; Fuchshuber and Unterrainer, 2021).

The *Leipzig Short Scale* (SOC-L9; Schumacher et al., 2000) based on the *Sense of Coherence Scale* (SOC; Antonovsky, 1993) measures SOC. The questionnaire contains nine items that are answered on a 7-point Likert scale. The ends of the scale are each anchored with different verbal response options. A higher score indicates a more pronounced SOC. Example items: “Do you feel that you are in an unfamiliar situation and do not know what to do?” (1 = *very often* to 7 = *very rarely or never*) and “You expect in the future that your own life will be” (1 = *without any meaning or purpose* to 7 = *full of meaning and purpose*). Satisfying internal consistency was found for the instrument (*α* = 0.84; Schumacher et al., 2000).

The short form (BSI-18; Derogatis, 2000) of the *Brief Symptom Inventory* (BSI; Derogatis and Melisaratos, 1983; German version by Spitzer et al., 2011) is a highly established instrument to assess psychological distress within the past 7 days. The inventory includes three subscales, Somatization, Depressiveness, and Anxiety, and has a total amount of 18 items that are rated using a 5-point Likert scale (0 = *not at all* to 4 = *very much*). In addition to the three subscales, a total score Global Severity Index (GSI) of all items can be calculated, which signifies the overall level of psychological distress. Examples of symptoms include “Feeling lonely” and “Spells of terror or panic.” The BSI-18 is a reliable and valid instrument (*α* = 0.93; Franke et al., 2017).

### Statistical analysis

Data were analyzed with IBM SPSS Statistics (Version 28). The items of the different scales were pooled into a total score for each measurement (involvement, psychological well-being, RSWB, SOC, psychological distress). Group differences in the potential control variables were analyzed with *t*-tests for independent groups for metric variables, Mann–Whitney-*U* tests for ordinal variables and chi-square tests of independence for categorical variables. Pearson correlations between the main variables were calculated and Bonferroni correction was applied to correct for multiple testing. Prior to conducting the mediation analyses, assumptions were checked including linearity, normality, homoscedasticity, multicollinearity, uncorrelatedness of residuals and absence of extreme outliers. To detect mediation effects of involvement on the relationship between practice type and psychological well-being, RSWB, SOC and psychological distress, the fourth model of the PROCESS macro (Hayes, 2022) was used, which calculates confidence intervals with a bootstrap method. The number of bootstrap samples was set at 5,000. For the exploratory analysis, *t*-tests for different reasons and Tukey-HSD for post-hoc comparisons were conducted. The rejection level for all analyses was set at *α* = 0.05. Tests were two-tailed.

A comparison of the two groups revealed a large age gap between the Yoga (*M* = 34.1, SD = 15.6) and Pilates group (*M* = 47.1, SD = 13.2), *t*(401.7) = −9.16, *p* < 0.001, *d* = −0.88, probably due to sampling bias because of convenience sampling. As the SOC-L9 is age dependent (Schumacher et al., 2000) and because age influences all dependent variables (Nilsson et al., 2010; López Ulloa et al., 2013; Moleiro et al., 2013; Dadfar et al., 2021) participants were matched by age to within 5 years. Therewith, we could ensure that both groups are comparable and avoid spurious effects due to age (Bland and Altman, 1994).

After matching, the Yoga and Pilates groups differed only in the potential control variables occupation (*χ*^2^ [7, *N* = 256] = 0.184, *p* = 0.01), psychiatric disorder (*χ*^2^ [1, *N* = 255] = 0.246, *p* = 0.003) and sport frequency (*U* = 7,011, *z* = −2.47, *p* = 0.01). Of those three, only occupation and psychiatric disorder were included as covariates because they seemed to be confounders, since they had the highest impact on most dependent variables. Occupation was related to SOC (Volanen et al., 2004), and to psychological well-being and psychological distress, especially during COVID-19 (OECD, 2021). Psychiatric disorders have a negative impact on psychological well-being, RSWB and SOC (Larsson and Kallenberg, 1996; Ristkari et al., 2008; Bartels et al., 2013; Unterrainer et al., 2014). They are closely intertwined with psychological distress, as psychological distress may signal disorders like depression or anxiety (Viertiö et al., 2021). However, one can experience psychological distress without the diagnosis of a psychiatric disorder. Because psychiatric diagnoses are more common among Yoga practitioners (Birdee et al., 2008) and because they often use Yoga to alleviate symptoms, we deemed it essential to include it as a covariate to reveal the effects of the different practices on psychological distress without the influence of preconditions.

Sport frequency was not accounted for, as higher levels in the Pilates group (compared to the Yoga group) could be an effect of practicing Pilates and, therefore, a downstream effect, not a confounder (Rosenbaum, 1984). Nine participants were excluded because of missing data in the covariates. After analyzing univariate and multivariate outliers, five more participants were excluded because they either appeared to be in a state of acute crisis (e.g., suicidal thoughts, panic attacks) or because they faced extreme demands (death and care of relatives as well as job loss). Normality was violated for psychological well-being (wide, left-skewed) and psychological distress (narrow and right-skewed). However, confidence intervals generated by bootstrapping do not require normality (Haukoos and Lewis, 2005; Pek et al., 2018). The scatter plot of standardized indicated a violation of the homoscedasticity assumption for psychological distress. Consequently, we applied the heteroscedasticity-robust standard error introduced by Davidson and MacKinnon to calculate that mediation (HC3; 1993), as it is the most reliable (Cribari-Neto et al., 2005).

## Results

### Sample and descriptive statistics

The final sample comprised 246 participants (216 women) whose ages ranged from 19 to 78 years (*M* = 45.0, SD = 15.0). Of those, 118 participants were in the Yoga group and 128 in the Pilates group. Table 1 displays socio-demographic variables for each condition and the differences between the groups. Pearson correlations between the main variables are displayed in Table 2.

**Table 1:** Sociodemographic data with group differences.

Baseline characteristic	Yoga (*n* = 118)			Pilates (*n* = 128)			*t*	*df*	*p*
	*M*	SD	Range	*M*	SD	Range			
Age	45.08	15.52	(19–78)	44.85	14.53	(19–78)	0.12	244	0.903
	*n*	*%*	*x*	*n*	*%*	*x*	*χ* ^2^	*df*	*p*
Gender							1.08	2	0.584
Female	101	85.6		115	89.8				
Country of residence							1.50	2	0.472
Germany	72	61.0		74	57.8				
Austria/Switzerland	46	39.0		54	42.2				
Highest education level							2.54	2	0.281
Secondary school degree^a^	20	16.9		13	10.1				
High school diploma^b^	32	27.1		37	28.9				
University degree	65	55.1		78	60.9				
Occupation							17.17	6	0.009
Apprenticeship	0	0		2	1.6				
Student	24	20.3		22	15.6				
Part-time^c^	57	48.3		51	39.8				
Full-time^d^	24	20.3		45	35.2				
Unemployed	5	4.2		0	0				
Homemaker	2	1.7		4	3.1				
Retired	6	5.1		6	4.7				
Relationship status							2.29	3	0.515
Single	24	20.3		18	14.1				
In a relationship	35	29.7		37	28.9				
Married	53	44.9		62	48.4				
Widowed/divorced	6	5.1		10	7.8				
Religious affiliation							0.99	4	0.912
No affiliation / non-believer	39	33.1		50	39.1				
Christianity (Roman Catholic)	30	25.4		34	26.6				
Christianity (Evangelical)	22	18.6		28	21.9				
Islam	3	2.5		2	1.6				
Other	8	6.8		7	5.5				

a Equivalent to EQF Level 2 and 3.

b Equivalent to EQF Level 4.

c 1–34 h per week.

d ≥35 h per week.

**Table 2:** Correlations between study variables (*N* = 249).

Variable	*α*	1	2	3	4	5
1. Y/P-S	0.93	—				
2. WHO-5	0.84	0.151*,^a^	—			
3. MI-RSWB 12	0.82	0.577***	0.284***	—		
4. SOC-L9	0.84	0.202**	0.595***	0.423***	—	
5. BSI-18	0.87	−0.088	−0.633***	−0.205**	−0.628***	—

*α* = Cronbach’s *α*; YI/PI-S = Yoga and Pilates Immersion Scale; WHO-5 = WHO-5 Well-Being Index (Psychological Well-Being); MI-RSWB 12 = Multidimensional Inventory for Religious/Spiritual Well-Being; SOC-L9 = Sense of Coherence Scale; BSI-18 = Brief Symptom Inventory (Psychological Distress). *p < 0.05. **p < 0.01. ***p < 0.001.

a Non-significant after Bonferroni correction.

### Mediation analyses

**Figure 1:**
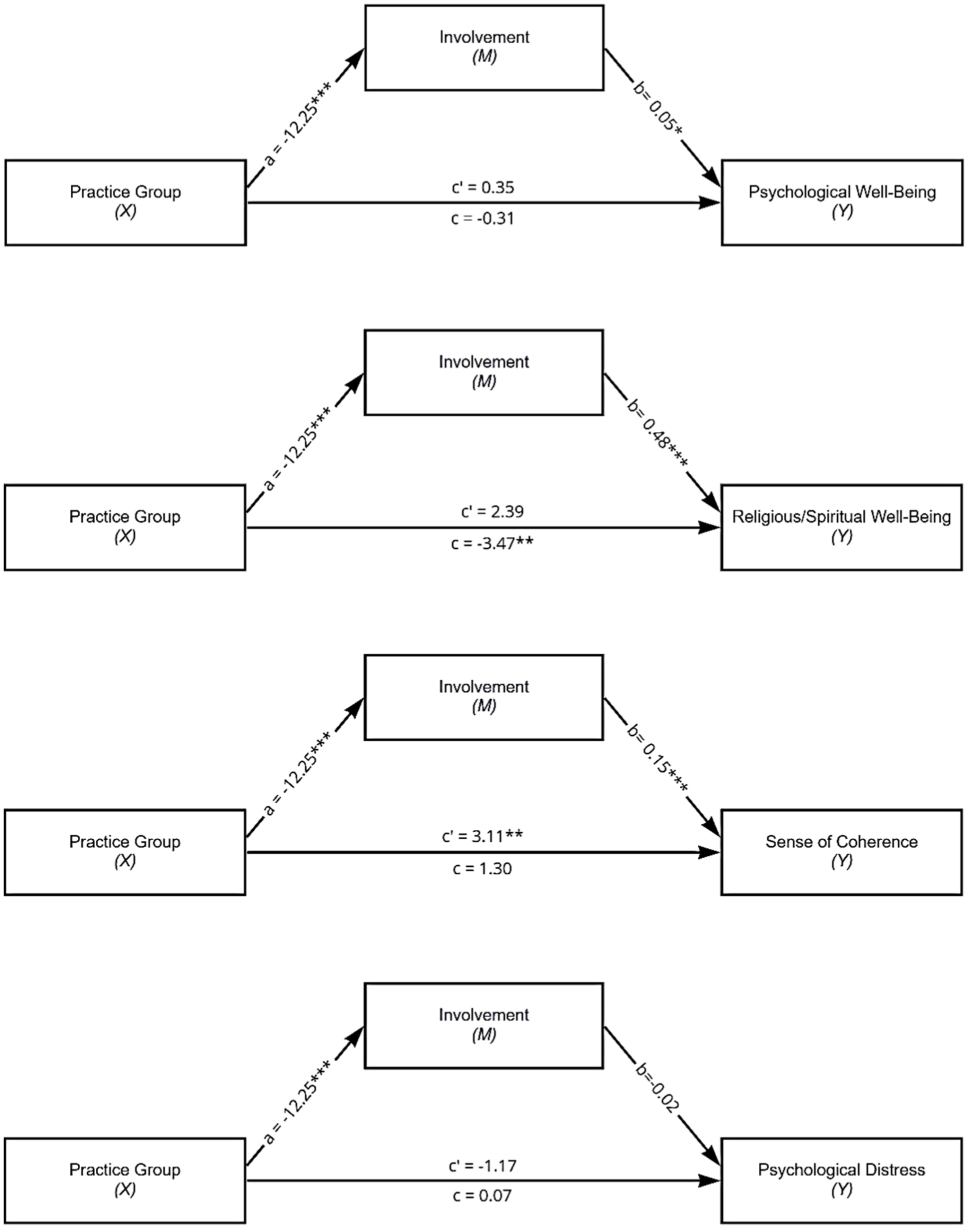
Mediation Models of the Influence of Involvement on the Relationship between Practice Group (Yoga or Pilates) and Psychological Well-Being, Religious/Spiritual Well-Being, Sense of Coherence and Psychological Distress. Standardized regression coefficients for the relationship between practice group and psychological well-being, religious/spiritual well-being, sense of coherence, psychological distress as mediated by involvement. *p < 0.05. **p < 0.01. ***p < 0.001.

The study was conducted to examine the effect of Yoga practice on different indicators of mental health as mediated by involvement in the practice. We hypothesized that practicing Yoga would positively affect involvement, which in turn positively affects psychological well-being, RSWB and SOC and negatively affects psychological distress. Table 3 and Figure 1 display the different mediation models.

**Table 3:** Mediation analyses of study variables.

Variable	*β*	SE	*t*	*p*	95% CI
Psychological well-being					
Total effect	−0.31	0.61	−0.52	0.606	[−1.51, 0.88]
Indirect effect	−0.66	0.32	–	–	[−1.32, −0.08]
Direct effect	0.35	0.68	0.51	0.611	[−0.99, 1.68]
Religious/Spiritual well-being					
Total effect	−3.47	1.32	−2.62	0.009	[−6.08, −0.86]
Indirect effect	−5.86	0.96	–	–	[−7.85, 4.14]
Direct effect	2.39	1.24	1.93	0.055	[−0.05, 4.83]
Sense of coherence					
Total effect	1.30	1.02	1.28	0.200	[−0.70, 3.31]
Indirect effect	−1.80	0.56	–	–	[−3.02, −0.78]
Direct effect	3.11	1.02	2.79	0.006	[0.91, 5.30]
Psychological distress					
Total effect	0.07	0.87	0.00	0.932	[−1.63, 1.778]
Indirect effect	0.24	0.42	–	–	[−0.534, 1.116]
Direct effect	−0.17	1.03	−0.16	0.873	[−2.199,1.867]

Standard errors and confidence intervals were derived from 5,000 bootstrapped models. Occupation and psychiatric disorder were included as covariates. Practice Group as the Independent Variable, Yoga and Pilates Involvement as the Mediator; and Psychological Well-Being, Religious/Spiritual Well-Being, Sense of Coherence, Psychological Distress as Dependent Variables. *p < 0.05. **p < 0.01. ***p < 0.001.

When controlling for presence of a psychiatric disorder and occupational status, Yoga practice positively predicted RSWB when compared to Pilates practice (*b* = −3.47, *t*(237) = −2.62, *p* = 0.01). However, contrary to expectations, it negatively predicted SOC (*b* = 3.11, *t*(236) = 2.79, *p* = 0.01). Yoga practice *per se* did neither predict psychological well-being nor psychological distress. Being part of the Yoga group predicted higher levels of involvement (*b* = −12,25, *t*(237) = −7,92, *p* < 0.001) than being part of the Pilates group. Involvement positively affected psychological well-being (*b* = 0.05, *t*(236) = 2.13 *p* = 0.03), RSWB (*b* = 0.48, *t*(236) = 10.35, *p* < 0.001) and SOC (*b* = 0.15, *t*(236) = 3.53, *p* < 0.001). It did not predict psychological distress.

When analyzing the mediation of involvement on the relationship between practice group and psychological well-being, neither the total (*b* = −0.31, *t*(237) = −0.52 *p* = 0.61) nor the direct effect were significant (*b* = 0.35, *t*(236) = 0.51 *p* = 0.61). However, the indirect effect was significant (−0.66, SE = 0.07, 95% CI [−0.28, −0.02]), which would indicate a mediation. Involvement mediated the relationship between group membership and RSWB (*ab* = −5.86, SE = −0.96, 95% CI [−7.95, −4.14]). The effect of Yoga practice on RSWB was not significant anymore when involvement was included in the model (*b* = 2.39, *t*(236) = 1.93, *p* = 0.55). This means that the positive relationship between Yoga practice and RSWB was fully mediated by involvement in the practice. For the putative relationship between practice group and SOC, the total effect was nonsignificant (*b* = 1.30, *t*(237) = 1.02, *p* = 0.20) but the mediated effect was significant (*ab* = −1.80, SE = −0.56, 95% CI [−3.02, −0.78]), which suggests an inconsistent mediation or suppression model (MacKinnon et al., 2000). According to that, Yoga practice would negatively influence SOC if involvement would not suppress its effect. Finally, there was no mediation of involvement for the relationship between practice group and psychological distress (*ab* = 0.24, SE = 0.42, 95% CI [−0.53, 1.12]).

### Exploratory analyses

In order to explore the cause for higher involvement in the Yoga group more deeply, we examined the participants’ reasons for starting the practice. Groups differed significantly in their primary motivation for starting the practice (*χ*^2^[12, *N* = 246] = 99.22, *p* < 0.001). Hereby, we found that Yoga practitioners were more likely to practice for spiritual reasons and for reasons more central to the practitioner’s self-construct (such as spirituality and finding oneself) when compared to the Pilates control group. Seven participants in the Yoga group stated that their primary motivation for starting the practice was spiritual, while none in the Pilates group did. Moreover, the reason for practice was significantly associated with levels of involvement (*F*(12, 246) = 4.99, *p* < 0.001). Post-hoc analysis revealed that individuals who practiced for spiritual reasons reported higher levels of involvement than people who practiced to better deal with health problems (*p* = 0.01), for flexibility (*p* = 0.004), movement (*p* = 0.03), fitness (*p* < 0.001), or fun (*p* =< 0.001). Individuals who practiced for self-discovery reported higher levels of involvement than people who practiced to better deal with health problems (*p* = 0.02), for flexibility (*p* = 0.01), movement (*p* = 0.05), or fitness (*p* < 0.001). The remaining differences were not significant.

## Discussion

This study was conducted to determine whether and how involvement mediates the effects of Yoga on different aspects of mental health, including psychological well-being, RSWB, psychological distress and SOC when compared to a Pilates control group. We expected more deeply involved individuals to experience increased mental health benefits. As predicted, the Yoga group was more involved in their practice than the Pilates control group, which endorses the assumption that a spiritual practice might lead to a deeper embeddedness than a non-spiritual practice (Gaiswinkler et al., 2015). Accordingly, participants in the Yoga group were more likely to start practicing for spirituality or self-discovery than participants in the Pilates group and practicing for these reasons was generally related to higher levels of involvement. Yoga as a practice, therefore, appears to be a more identity-forming than Pilates and seems to profoundly shape how a practitioner experiences the world.

### The relationship between yoga and mental health

In contrast to our assumptions, the Yoga group did not experience higher levels of **psychological well-being** than the Pilates group. However, Yoga practitioners were more immersed in their practice, which in turn was associated with an increased well-being. This is in line with general findings that Yoga facilitates well-being in general (Woodyard, 2011), and in the pandemic especially (Sahni et al., 2021). One explanation for the insignificant overall effect might be that the study did not have enough power to detect an overall effect, and a larger sample would have yielded a mediation with a significant overall effect (Kenny and Judd, 2014).

As expected, involvement mediated the positive relationship between Yoga practice and RSWB. We even found a full mediation, explaining indicating that involvement is the key factor in explaining the higher levels of RSWB in the Yoga group. Consistent with our model and prior research, these results suggest that involved Yoga practitioners experience higher levels of RSWB (Gaiswinkler and Unterrainer, 2016) than Pilates practitioners. This is probably because they engage in a spiritual practice resulting in higher involvement, which in turn elevates RSWB. Consistent with this are also the findings that an intense practice deepens the spirituality of Yoga practitioners (Büssing et al., 2012a). In the secularized West, where religious practice and identification have waned (Molteni and Biolcati, 2018), a spiritual practice such as Yoga may provide similar mental health benefits (see Csala et al., 2021 for further discussion). Contrary to expectations, Yoga practice was associated with a lower SOC than Pilates practice but with higher levels of involvement, and with because involvement increased SOC, the potential negative effect of Yoga practice on SOC was suppressed by involvement. Our exploratory findings provide some insight into these unexpected results, since we discovered that yogis and yoginis practice more frequently with the intention of fostering spirituality and finding themselves. Yoga practitioners are more likely to suffer from depression and anxiety (Birdee et al., 2008), which are linked to a low sense of coherence (Carstens and Spangenberg, 1997). People who start to practice Yoga for these reasons may lack sense of coherence and wish to compensate for it. Perhaps then, because Yoga practice has been associated with an increasing increased sense of coherence (Ando et al., 2011; Gwiaździński et al., 2017), Yoga’s propensity for higher involvement may have buffered preexisting negative levels of sense of coherence. On the other hand, true causal effects of practice differences may be reflected. Hereby, either Pilates improves sense of coherence more than Yoga does or Yoga diminishes sense of coherence, since meditative states achieved by Yoga practice might mirror some aspects of dissociative states (Waelde et al., 2004), which could impair a sense of comprehensibility or meaningfulness.

Contrary to previous research, Yoga practice was not significantly associated with lower levels of **psychological distress**. Yoga was associated with increased levels of involvement, but higher involvement did not lead to lower levels of psychological distress. Although we controlled for psychiatric disorders, it is entirely possible that yoga practitioners may have higher levels of psychological distress without meeting the threshold for a diagnosis, as vulnerable individuals may choose yoga to soothe their symptoms (Orlygsdottir et al., 2021). If Yoga practice and, more specifically, Yoga involvement alleviates symptoms and anxiety as expected (Li and Goldsmith, 2012; Cramer et al., 2013; Gaiswinkler and Unterrainer, 2016; Cramer et al., 2018), both effects might cancel each other out. Possibly, as suggested by Gaiswinkler and Unterrainer (2016), only highly involved practitioners experience relief of psychological distress.

### Limitations and future research

The biggest limitation of the study is the correlative, cross-sectional design, which restricts conclusions regarding causal relationships (van der Stede, 2014). It would be possible, too, that people who experience higher levels of well-being find it easier to involve themselves in their practice, not vice versa (Gaiswinkler and Unterrainer, 2016). We did, however, compare the Yoga group to an age-matched control group and examined several possible confounders to have higher confidence in the directionality of our findings. To obtain more accurate measures, future studies could additionally consider the different styles of Yoga and Pilates, as well as status as a teacher or student.

Furthermore, data were obtained solely through self-assessment, allowing participants to partake from home with minimal time commitment and risk of being exposed to COVID-19 but possibly inducing common-method bias. For a follow-up study, a longitudinal design where biological parameters (e.g., cardiovascular activity or imaging techniques) are considered would be highly desirable (Balasubramaniam et al., 2012). Randomly assigned groups instead of convenience sampling would increase internal validity and ensure that groups do not differ as greatly in age (Joshi and Singh, 2020). To establish a causal relationship, it would be vital to obtain two measurements (pre-and post-treatment) and calculate the difference between them. The Pilates control group was more homogenous than in previous research, single-blinded intervention involving some sort of “quasi-Yoga,” that does neither include spirituality, nor breath control might help more carefully analyze specific aspects of Yoga. However, it might be challenging to isolate basic elements of the practice (Balasubramaniam et al., 2012).

Finally, future research could explore what exactly causes higher levels of involvement besides the reason for starting the practice. The relationship with the instructor, a sense of community, private versus public practice, different personality traits (especially conscientiousness, openness, and neuroticism), self-compassion, and/or mindfulness may be of particular interest (Gaiswinkler et al., 2020). Since involvement predicts various indicators of well-being, further research could alter these possible predictors of involvement to intervene and heighten involvement. Involvement might even be useful in a clinical setting, where facilitating an involved practice could promote established positive effects of Yoga on depression, anxiety, trauma, somatic symptom disorders and schizophrenia (Forfylow, 2011; Balasubramaniam et al., 2012; Nguyen-Feng et al., 2019; Simon et al., 2021; Tibbitts et al., 2021), thereby serving as a therapeutic adjunct in line with traditional psychotropic medication and psychotherapy.

## Conclusion

In this study, Yoga involvement could be further confirmed as a useful construct to explain how Yoga practice influences various states of mental health and illness. As expected, involvement was higher in the Yoga group and predicted several indicators of well-being. A full mediation was only found for RSWB, while involvement buffered potential negative effects of practicing Yoga on SOC. Yoga and involvement may not be the panacea for all varieties of psychological difficulties caused by current stressors. However, both could provide an important piece of the puzzle, as part of a holistic clinical treatment approach.

## Data availability statement

The raw data supporting the conclusions of this article will be made available by the authors, without undue reservation.

## Ethics statement

The studies involving human participants were reviewed and approved by Ethics Committee of the University of Graz. The patients/participants provided their written informed consent to participate in this study. Written informed consent was obtained from the individual(s) for the publication of any potentially identifiable images or data included in this article.

## Author contributions

CB and H-FU conceptualized the study. CB collected, analyzed and interpreted the data and drafted the first version of the manuscript. H-FU supervised the writing of the manuscript. JF, LG, and AS critically reviewed the manuscript. All authors contributed to the article and approved the submitted version.

## Conflict of interest

The authors declare that the research was conducted in the absence of any commercial or financial relationships that could be construed as a potential conflict of interest.

## Publisher’s note

All claims expressed in this article are solely those of the authors and do not necessarily represent those of their affiliated organizations, or those of the publisher, the editors and the reviewers. Any product that may be evaluated in this article, or claim that may be made by its manufacturer, is not guaranteed or endorsed by the publisher.

## Footnotes

^1^The participant’s consent to publish their statement was obtained.
